# Designing a Multimodal and Culturally Relevant Alzheimer Disease and Related Dementia Generative Artificial Intelligence Tool for Black American Informal Caregivers: Cognitive Walk-Through Usability Study

**DOI:** 10.2196/60566

**Published:** 2025-01-08

**Authors:** Cristina Bosco, Ege Otenen, John Osorio Torres, Vivian Nguyen, Darshil Chheda, Xinran Peng, Nenette M Jessup, Anna K Himes, Bianca Cureton, Yvonne Lu, Carl V Hill, Hugh C Hendrie, Priscilla A Barnes, Patrick C Shih

**Affiliations:** 1 Luddy School of Informatics, Computing, and Engineering Indiana University Bloomington, IN United States; 2 School of Nursing Indiana University Indianapolis, IN United States; 3 Alzheimer's Association Chicago, IL United States; 4 School of Medicine Indiana University Indianapolis, IN United States; 5 School of Public Health Indiana University Bloomington, IN United States

**Keywords:** multimodality, artificial intelligence, AI, generative AI, usability, black, African American, cultural, Alzheimer's, dementia, caregivers, mobile app, interaction, cognition, user opinion, geriatrics, smartphone, mHealth, digital health, aging

## Abstract

**Background:**

Many members of Black American communities, faced with the high prevalence of Alzheimer disease and related dementias (ADRD) within their demographic, find themselves taking on the role of informal caregivers. Despite being the primary individuals responsible for the care of individuals with ADRD, these caregivers often lack sufficient knowledge about ADRD-related health literacy and feel ill-prepared for their caregiving responsibilities. Generative AI has become a new promising technological innovation in the health care domain, particularly for improving health literacy; however, some generative AI developments might lead to increased bias and potential harm toward Black American communities. Therefore, rigorous development of generative AI tools to support the Black American community is needed.

**Objective:**

The goal of this study is to test Lola, a multimodal mobile app, which, by relying on generative AI, facilitates access to ADRD-related health information by enabling speech and text as inputs and providing auditory, textual, and visual outputs.

**Methods:**

To test our mobile app, we used the cognitive walk-through methodology, and we recruited 15 informal ADRD caregivers who were older than 50 years and part of the Black American community living within the region. We asked them to perform 3 tasks on the mobile app (ie, searching for an article on brain health, searching for local events, and finally, searching for opportunities to participate in scientific research in their area), then we recorded their opinions and impressions. The main aspects to be evaluated were the mobile app’s usability, accessibility, cultural relevance, and adoption.

**Results:**

Our findings highlight the users’ need for a system that enables interaction with different modalities, the need for a system that can provide personalized and culturally and contextually relevant information, and the role of community and physical spaces in increasing the use of Lola.

**Conclusions:**

Our study shows that, when designing for Black American older adults, a multimodal interaction with the generative AI system can allow individuals to choose their own interaction way and style based upon their interaction preferences and external constraints. This flexibility of interaction modes can guarantee an inclusive and engaging generative AI experience.

## Introduction

### Background

Alzheimer disease and related dementias (ADRD) are brain health conditions impacting cognitive abilities such as memory, language, problem-solving, and executive functions [1,2]. Within the United States, older Black American and Black adults have been identified as being twice as likely as individuals from other ethnic groups to develop ADRD over the course of their lifetime [3-5]. In a study conducted with 59,555 individuals, Black American adults had a 65% greater risk of developing ADRD (with 26.6/1000 Black American adults developing ADRD, compared with rates of 19.6/1000 for Latinos, 19.6/1000 for Pacific Islanders, 19.3/1000 for Whites, and 15.2/1000 for Asian Americans) [6].

However, despite being significantly more likely to develop ADRD, Black Americans exhibit lower levels of ADRD-related health literacy compared with other demographic groups [7]. This disparity impacts informal caregivers who often find themselves unprepared and uninformed about ADRD treatment and diagnosis, as well as about ADRD-related sources and help [8]. Therefore, providing essential medical information to these caregivers is crucial in combating the disease.

Considering the potential of new technologies to aid patients and caregivers with understanding medical language by simplifying content and removing jargon [9], generative artificial intelligence (AI) tools, like ChatGPT developed by OpenAI, could offer innovative opportunities in health care, particularly for improving ADRD-related health literacy. Indeed, recent studies proposed using ChatGPT for reliable and comprehensive health information [10-13].

However, relying solely on generative AI to improve underrepresented communities’ health literacy can lead to several issues like providing inaccurate or false health information [14,15]. Additionally, studies have shown that using generative AI exclusively for health diagnosis and treatment can reinforce sexist and racial stereotypes and biases [16-18].

For instance, predictive models trained on historical data that lack representation from diverse demographics can reinforce existing biases in diagnosis and treatment plans [19,20]. The exclusion of certain populations in the studies and design process can lead to misdiagnosis and inappropriate treatment [21-23], exacerbating health inequalities rather than reducing them [20,24,25]. Therefore, it is crucial to ensure greater diversity in model training data and include diverse participants in studies and design processes to improve the generalizability of findings [26] and the fit of the technology use for the target population.

Therefore, given the potential of generative AI to improve health literacy and mitigate health inequalities among underrepresented populations, such as the Black American community, it is imperative to design inclusive ADRD health literacy interventions.

Previous studies, especially in the domain of human-centered design, highlight the importance of integrating cultural insights into health care technologies for this community [27-31]. Hence, it is crucial to tailor generative AI–based health interventions to meet the unique needs of Black American informal caregivers of individuals with ADRD.

Thus, this study identified the needs of Black American caregivers of individuals with ADRD by testing Lola, an ADRD-focused generative AI tool. Lola is a multimodal mobile app that provides access to health information from the Alzheimer’s Association online repository [1] via text, chatbot, and voice assistant.

### Related Work

#### Generative AI in Health Care

Generative AI has become a transformative force in health care, offering innovative solutions in diagnostics, personalized medicine, and patient care. Recent studies have highlighted the role of generative models in enhancing diagnostic accuracy by analyzing complex medical data and generating synthetic patient data to train other AI systems. For example, a study by Chen and Esmaeilzadeh [32] demonstrated that generative models like generative adversarial networks could significantly improve the detection and diagnosis of diseases by creating high-quality medical images from existing data. Additionally, variational autoencoders have been used to generate realistic medical imaging data, further aiding disease research and model training. Moreover, the use of large language models (LLMs) such as GPT-4 has shown potential for assisting clinicians by generating comprehensive patient histories and suggesting personalized treatment plans [33].

Moreover, the use of LLMs such as generative pretrained transformer (GPT)-4 has shown potential for assisting clinicians by generating comprehensive patient histories and suggesting personalized treatment plans [33]. Studies have also explored the use of LLMs in generating patient education materials and automating routine documentation tasks [34]. Despite still being in its developmental stages, generative AI holds significant promise for enhancing the accessibility of health care [10-13].

#### Human-Centered Generative AI

Generative AI models are trained on large data sets that may contain biases, further perpetuating stereotypes and disparities in health care [20,24]. More diversity is needed in both data used to train AI models and participants included in studies to ensure the generalizability of findings [26]. Additionally, addressing ethical and regulatory considerations is crucial when deploying AI in health care.

The integration of human-centered design principles into generative AI applications is crucial to ensure these technologies are accessible, intuitive, and beneficial to end users. Research has emphasized the importance of involving end users in the development process to create AI systems that meet their needs and preferences. For instance, a study explored the application of ChatGPT for developing digital health interventions, finding that user involvement in the design phase led to higher satisfaction and engagement rates among patients [35]. Specifically, user feedback was gathered through focus groups and iterative testing phases, allowing developers to continuously refine the AI user interface and functionality.

#### Generative AI and the African American and Black Community

Inequalities have been observed in health care and accessibility to these services for underrepresented communities [36-40]. AI systems that initially aim to reduce the disparities in health care also present challenges for health care accessibility for Black American communities.

Addressing the unique health care challenges faced by Black American communities through generative AI is a growing area of interest. It is essential to ensure that these AI systems do not perpetuate existing biases or disparities. Studies have highlighted the potential of generative AI to provide culturally sensitive health care solutions. For example, a study explored the use of mobile health [41] interventions to mitigate the impact of COVID-19 in Black American communities, emphasizing the need for community-specific content and trusted communication channels to improve health outcomes.

#### Informal Caregivers of Individuals With ADRD and Their Use of Technology

In 2023, approximately 15 million people served as caregivers for individuals with ADRD [1], with African Americans accounting for one-tenth of these caregivers [1]. This unpaid work amounted to an estimated 16 billion hours [1]. Caregivers often feel unprepared for their roles [42], lacking information on diagnosis, treatment, and available resources [8,43].

Technology has been crucial in supporting these caregivers [44], and technologies have been developed to reduce caregiver stress and anxiety [45] and facilitate access to information for caregivers [46]. However, these technologies targeted different demographics than African American and Black populations. For instance, Hong et al [45] developed and tested a technological intervention aimed at reducing the stress and anxiety related with caregiving by focusing exclusively on Chinese participants. On a similar note, Boutilier et al [46] developed and tested a web app to help informal caregivers document information on their ADRD-diagnosed patients and share important changes with the care team. Boutilier et al [46] tested this system with mainly non-Hispanic White participants, with only 1% of participants being Black Americans.

However, no study has focused on Black American caregivers, which is extremely important and challenging at the same time, considering this population’s lower level of technology use and adoption [47]. Additionally, a recent systematic review of technological interventions (mainly mobile apps) [48] found that most of these systems did not present any culturally relevant health information and were characterized by low accessibility and low readability.

Previous studies have shown that online health communities (OHCs) offer emotional and instrumental support to caregivers of individuals with ADRD [49] and chronic diseases [50], providing a platform to share experiences and find support from peers [49,51]. Furthermore, only a few technological support interventions for informal caregivers focus on ADRD-related information. Most aim to enhance caregivers’ psychological well-being by providing stress management strategies [52] or developing social games to promote physical activity and mutual support networks [53].

We identified 2 gaps in the previous literature. Although OHCs offer instrumental support by sharing caregiving tips, they often lack localized, actionable ADRD information or personalized support tailored to caregivers’ and patients’ specific needs. Additionally, there is limited representation of Black American caregivers in research, raising concerns about the ability to understand and address their sociocultural background as well as their unique needs compared with other populations, which is vital for developing health technologies for them [49].

## Methods

### Recruitment

We conducted recruitment and research sessions in close collaboration with a community engagement team who ensured that participants were all caregivers in some capacity for individuals living with ADRD and fulfilled the inclusion criteria. To meet the inclusion criteria, participants had to be older than 50 years, identify as Black Americans, reside in the target area, and actively provide care for a person with ADRD. Throughout this study, emphasis was placed on collaboration with the Black American community within the region. Consequently, 2 community engagement specialists joined the research team to organize monthly community advisory team board meetings to maintain community involvement and awareness of research insights and findings. Prior to implementing any major design decisions, the advisory team board was consulted to ensure cultural relevance. These measures enabled the research team to make more culturally competent decisions.

### Ethical Considerations

Ethical approval was provided by the institutional review board at Indiana University (approval number 12241), All participants received a full explanation of the consent procedures, and any specials needs were accommodated during sessions. Participants were compensated with a US $50 gift card for taking part. Participants’ privacy and confidentiality were protected by anonymizing the data, changing the wording of the quotes presented in the Results section, and deleting all the audio recordings after the transcriptions were completed.

### Data Collection and Analysis

We performed an adapted cognitive walkthrough with users [54,55]. We conducted 15 sessions in 2 regions in the Midwest region of the United States. Each session was completed in a 1-on-1 meeting that involved 1 researcher and 1 notetaker with the participant. Each session lasted approximately 30 minutes. Although we were aware that longer testing sessions might guarantee the collection of more comprehensive feedback, we made sure to construct the tasks and testing in a way that allowed the emergence of in-depth insights and findings. Moreover, given the older age of our participants, we did not want to induce cognitive fatigue.

During a session, each participant was introduced to the platform and a set of 3 tasks. Those 3 tasks consisted of searching for an article about brain health, searching for ADRD-related local events, and finally, searching for opportunities to participate in scientific research.

As participants performed the tasks, they were asked to voice their thoughts out loud in real time (“think aloud” method [56]), and after each task was complete, there was a brief semistructured discussion centered around 4 tenets: (1) usability (eg, Did you find anything in this task confusing? In what way?), (2) accessibility (eg, How do you expect Lola to answer you? Did you find the information easy to understand? If not, why?), (3) cultural relevance (eg, Did you find the tone of interactions appropriate? If not, how so? How do you think this platform can be made better for the Black American community?), (4) adoption (eg, What do you think would be a good way of spreading knowledge about this so that people you know can properly adopt it and use it?)

Each session was audio-recorded and transcribed. The generated transcripts were spell-checked. Guided by the principles of usability, accessibility, and relevance, 6 researchers coded the transcripts using the open-source software Taguette [57]. The team took an analytical, iterative approach by discussing the codes and consolidating them into themes [58] through second and third rounds of discussion. The resulting major themes about the needs of the users are described in the Results section.

### System Design

#### Overview

Generative AI is at the forefront of technological advancements in health care applications, specifically in the development of interactive tools such as chatbots and voice assistants. In the context of Lola, an innovative app tailored for Black Americans coping with ADRD, generative AI plays a crucial role. It powers both the chatbot and voice assistant, enabling them to deliver intuitive and empathetic user interactions. These AI-driven tools analyze complex user queries, source relevant information, and articulate responses that are both informative and comforting.

#### Integration With ChatGPT Technology

The core functionalities of Lola—the chatbot and voice assistant—use the natural language processing prowess of ChatGPT 3.5 Turbo.

#### Voice Assistant and Chatbot Mechanism

These multimodal features enhance the accessibility of the app, providing users with options for both auditory and textual communication. This is particularly beneficial for users with different levels of technological literacy or physical abilities. The real-time response generation capability of ChatGPT ensures that every interaction is smooth and engaging.

#### Data Utilization and Information Sourcing

Lola’s AI systems are designed to draw information from authoritative sources such as the Alzheimer’s Association online repository [1], supplemented by curated recommendations from a team of medical doctors specializing in ADRD. This method ensures that the information provided is not only accurate but also up to date with current medical standards and practices.

#### Ensuring Clear and Meaningful Communication

The generative process involves a sophisticated workflow in which initial responses, structured using LLM techniques, are refined by ChatGPT for clarity and empathy. This ensures that the communication is not only technically accurate but also tailored to meet the emotional and cognitive needs of the users.

#### Technical Infrastructure

The comprehensive technical infrastructure of Lola uses Flutter for development of the front end to maintain a consistent and engaging user experience with visually appealing, high-performance interactions across various devices and modalities. The back end is powered by Python’s Flask framework, paired with a PostgreSQL database for robust data management and fast data retrieval during the human-app interactions. The deployment of Docker for containerization, along with the use of the research team’s university servers, provides a secure and reliable environment for hosting and managing Lola’s functionalities. By reducing compatibility issues, this setup ensures consistent system performance and reliability.

#### App Features

**Figure 1:**
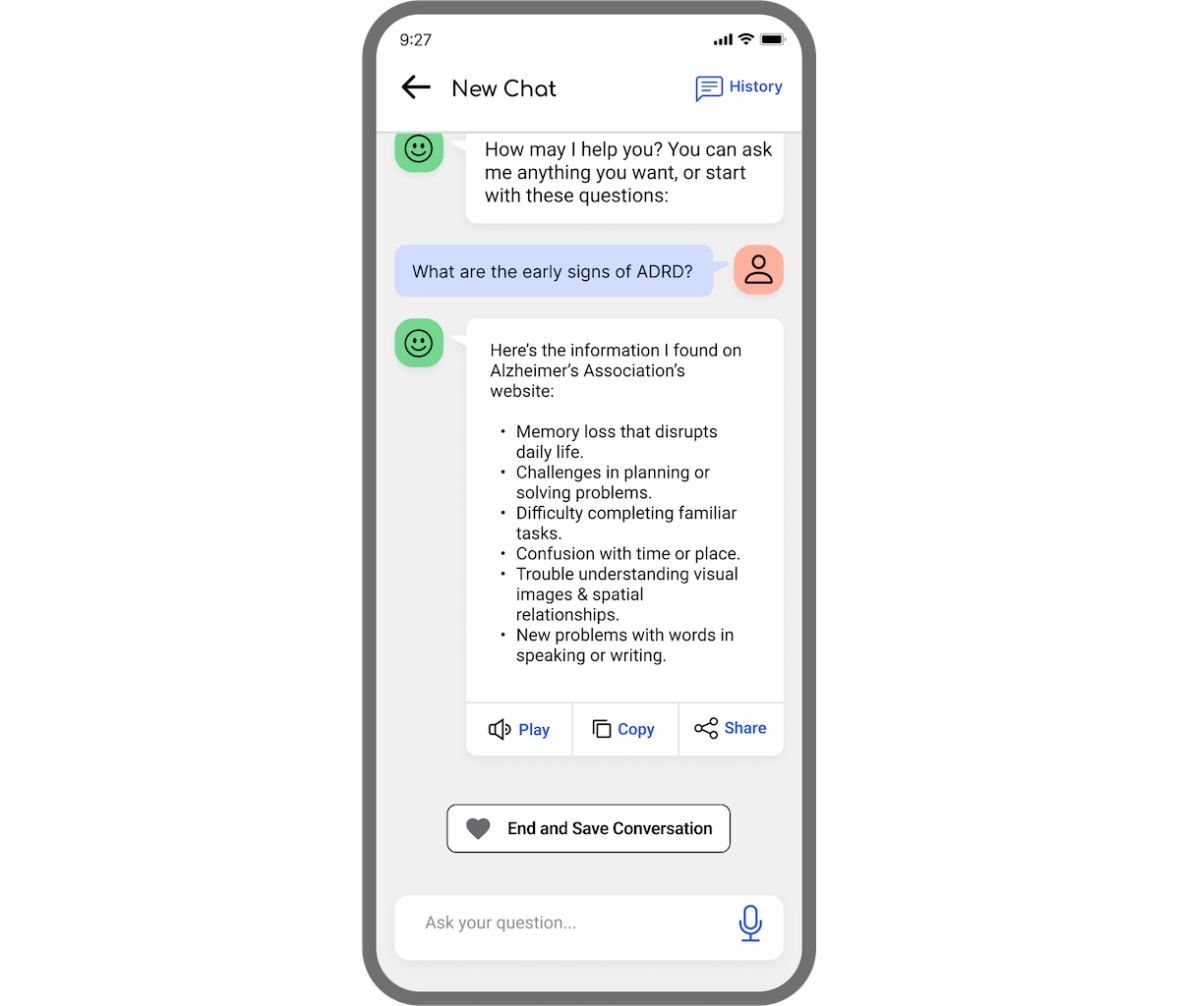
Example of a user having a conversation with the Lola chatbot using the interface designed by the research team.

In response to the challenges faced by older Black Americans regarding the accessibility of credible ADRD information, Lola’s interface serves as a platform for accessing ADRD-related articles, local and virtual events, and opportunities to engage in the latest ADRD research. Lola incorporates a chatbot feature represented by a floating green bubble in the bottom right corner, accessible to users throughout their interaction with the app (Figure 1).

**Figure 2:**
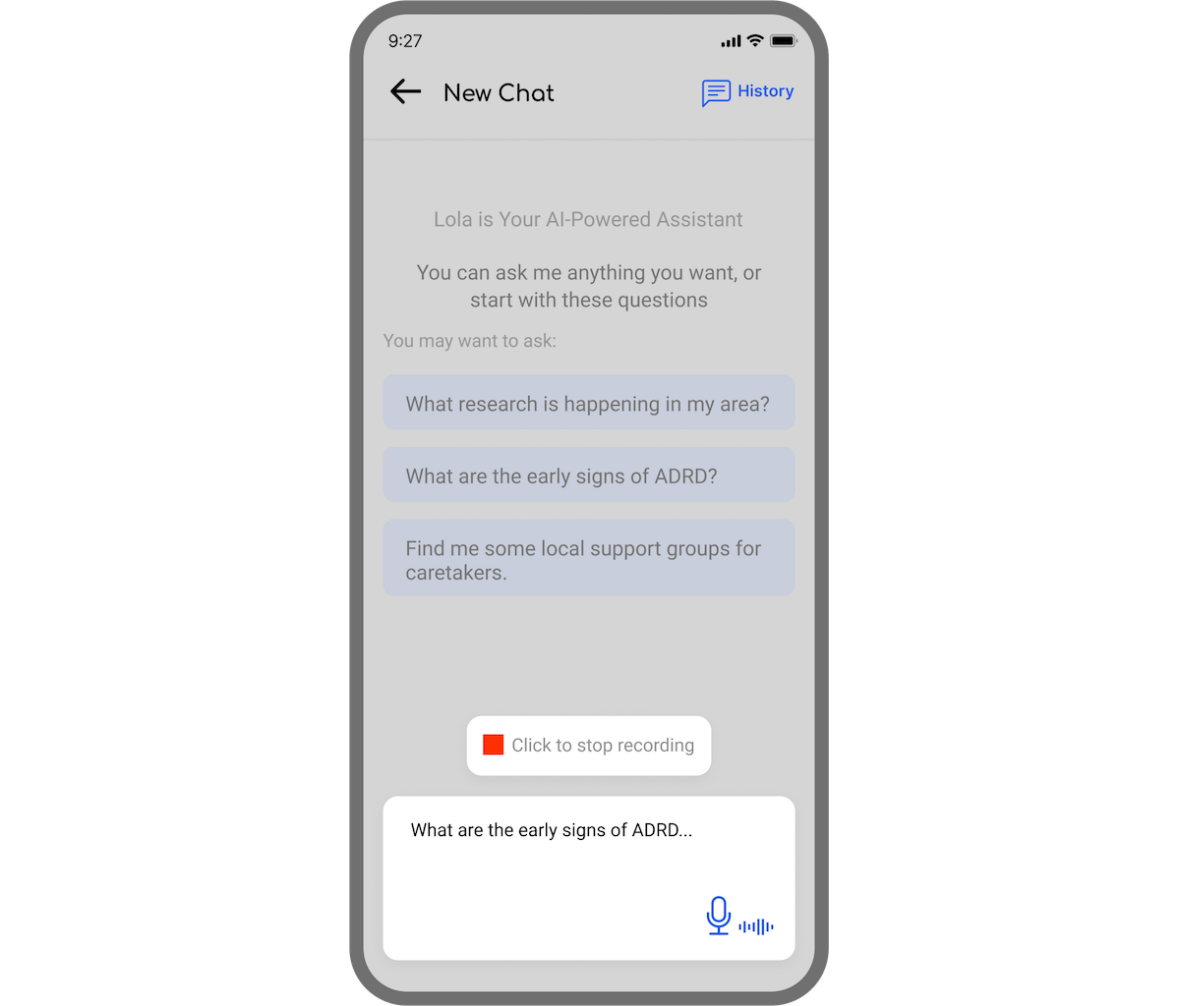
Example of a user using voice input and the possibility of browsing the history.

Users can interact with the Lola chatbot by clicking the green call-to-action button, which initiates a conversation through text or voice input. Using generative AI, the Lola chatbot provides responses in text format or as voice messages by clicking the “Play” button, facilitating accessibility for users with varying literacy levels or preferences for voice interaction. Users can also copy the response as text or share the response with their contacts. When users choose to end the conversation, the system saves the conversation in the chat history (Figure 2).

## Results

### Participant Demographic Information

A total of 15 participants were included in this study. All of them were informal caregivers for someone living with ADRD and were Black American. All participants shared that they were born and raised in the United States. Regarding family origins, 5 participants identified having African family origins, 9 were unsure of their family origins, and 1 chose not to disclose. Table 1 summarizes the participants’ demographic data. Of the 15 participants, 12 (80%) were 65 years or older. Most participants were female (11/15, 73%), with 2 (2/15, 13%) being male and 2 (2/15, 13%) providing no gender response. Regarding education, 10 participants (10/15, 67%) indicated having at least some college education (partial completion, a bachelor's degree, or an associate degree), while 2 (2/15, 13%) had a graduate degree, 2 (2/15, 13%) indicated a high school education, and 1 (1/15, 7%) did not provide a response. Most participants (11/15, 73%) were retired, only 3 participants (3/15, 20%) worked either part-time or full-time, and 1 (1/15, 7%) did not respond.

**Table 1:** Participant demographic information.

Participant number	Age (years)	Gender	Education level	Employment	Work sector
1	72	Female	Some college	Retired	Health care
2	74	Male	Some college	Retired	Utilities
3	68	Female	Associate’s degree	Retired	NR^a^
4	69	Male	Some college	Retired	Construction
5	54	Female	Associate’s degree	Part-time	Health care
6	66	NR	NR	NR	NR
7	69	Female	Graduate degree	Retired	Community advocacy
8	69	Female	Some college	Retired	Health care
9	75	Female	Some college	Retired	Faith-based
10	71	Female	High school	Retired	Customer service
11	69	Female	Associate’s degree	Retired	Insurance
12	72	NR	High school	Retired	Health care
13	68	Female	Some college	Retired	Health care
14	63	Female	Graduate degree	Full-time	Employment specialist
15	62	Female	Bachelor’s degree	Part-time	Education

^a^NR: not reported.

### Overview of Lola

The Lola system includes 3 different models of interaction: (1) accessing information in a traditional touch screen app format, (2) texting with an AI-powered text-based chatbot, and (3) talking to a voice assistant that accompanies the chatbot. Participants experienced all 3 models of interaction during user testing.

### Themes

From the analysis of the data collected, 3 main themes emerged: (1) the need for a multimodal AI tool that relies on multiple senses, (2) the need for culturally specific ADRD content, and (3) the importance of physical places and existing social networks to spread Lola among the community. This section will explore these themes in depth and how they relate to the goals of the research.

#### Theme 1: The Need for a Multimodal AI Tool That Relies on Different Senses

This theme describes participants’ need for interacting with the Lola system in different ways aside from the traditional touch screen gesturing predominant in most mobile apps. This multimodal interaction could take several forms, for example, voice interaction that can read content generated as text out loud. This would allow participants with low vision, which is common among older adults, to access the content.

Because with the people that I work with on a daily basis, one is not able to read anymore because she’s going blind. So, it’s not just Alzheimer's dementia that needs to be looked at, at least all the senses need to be looked at because people are going through so many different things at the same time.

P4

Consistently, auditory access to information positively influenced participants’ interactions with the system. Some participants preferred to engage with the tool using their voice, hence treating the tool as a voice assistant and asking it questions. For the participants, this was a valuable addition to the traditional touch screen interaction. For people not only with low vision but also with lower digital skills, voice interaction resembles a conversational exchange with which they are familiar and comfortable.

I think that this was good to be able to speak it. Because, if you’re dealing with people who are not computer savvy or internet savvy, being able to speak it, and it automatically pops up or speaks your question and then being able to close that area so it won’t keep recording. That was quick and easy. It was simple.

P9

Speaking features (as opposed to just reading and typing information) are especially helpful since they would enable people to access their usual methods of dealing with health-related information, such as writing it down on a piece of paper and saving it for later reference while someone is sharing the information. Having an auditory feature to provide health care information in addition to other interaction models can improve the accessibility of relevant information for those with difficulties accessing digital content due to low visual capacity.

That would help too if it played. And then they spoke out the information. And there’s different ways I can have a pen and a pad. And then while she’s speaking, I could write the information down. Because it is printed small. And you have to read all this information. But if she tells me, and the number you can call is one 800, I’d be writing it down.

P1

However, solely relying on the sense of hearing could be a limitation for people with auditory impairments, who could get frustrated by the tool.

But I couldn’t hear it. I was kind of frustrated. So, it kind of forced me to read it in my eyes are strained.

P14

One participant highlighted the benefit of a multimodal approach pairing visual and auditory stimuli together, drawing on their experience in church where reading and listening occur simultaneously.

For me and people lazy, they don’t want to have to go through the reading it would have, because I would like to hear it while I’m reading it. It is better. If it were just speaking it, I probably wouldn’t grasp. But it was speaking in I was reading it and I was falling through. It would have been like being in church. And the preacher is preaching a sermon, he tell you what he coming out of and you fall you open your Bible to that. John 316, and you follow him as he’s talking.

P5

This theme highlights the importance of creating a system with multimodal interaction and pairing auditory and visual inputs in a way that makes the tool more accessible and allows a broader audience to meaningfully engage.

#### Theme 2: The Need for Context and Culture-Specific ADRD Content

This theme highlighted the need for contextually and culturally specific mobile apps that integrate relevant and accessible ADRD information. When combined with a multimodal AI tool, these tailored apps can significantly enhance participants’ access to the information they need, which is not always directly related to health care.

One participant expressed their discontent with the lack of relevant information in the Lola app, stating that they wanted more than just medical information such as actionable lifestyle changes that can improve their health.

Definitely more than just medical information. Because I’m not one to push drugs, I don’t take none. If a doctor tells me something is going on, I’m asking first and foremost, what can I do? Or how can you help me get to this process? Naturally? Do I need to implement exercise, do I need to change my diet, what vitamins, whatever. But if they talk about medication, it’s gonna be a big turn off for me instantly. So that's just me. Some people think a quick fix is the answer. But every Quick Fix has consequences.

P4

Additionally, another participant mentioned the need for accessible ADRD mobile apps. Being able to operate and navigate through the app with little or without outside help is ideal.

Well, you know I just had therapy at the hospital, okay. And I was so amazed that every time I went to therapy, they gave me a folder with exercises, and one time they gave me an app. A girl was there, and she showed that to me. And I was just shocked. She used the app to show me the exercises, and every time I went back, she added exercises for me to do on the app. So that was amazing to me. So yeah, the apps are important. They really are. And they're to be simple. And they got to be informative. And they got to be to the point where even the agent can navigate through them.

P10

Some participants expressed discontent with the use of language in the Lola app, as it was perceived as too technical and not catering to the target population well enough.

Now all these big words, that’s what we get me. I wouldn’t understand them. Okay, so there’s too many words, too many big words, I would not understand.

P15

Certain participants expressed a desire for the mobile app to cater to their Black American communities by offering culturally specific ADRD information tailored to their needs. Consequently, they sought additional content that would address their unique concerns and circumstances.

So I’m thinking on the other side as the creators of it, maybe you don’t want to offend other races or whatever. But if Alzheimer’s is more prevalent in my community, I feel like we need to have that target. So we will know exactly where to go, filter through some of the other stuff, some of the things but Alzheimer’s and dementia are across the board. But maybe some things are just specific toward my race. Sure. And I want to know that, and I want to know that in the most timely fashion possible.

P9

Last, some of the participants highlighted the necessity for the mobile app to include community-centric information and information about local resources and organizations for ADRD, which could be tailored to individual users and their community based on their location.

I don’t travel too far for it. So, I think there needs to be something located in their community.

P4

More websites because a lot of people are interested in a study of Alzheimer’s or dementia. And it’s national, local. So, I would rather you know, her give me the local if I had to visit it. It’s good to know the national, but also there’s local organizations as well.

P1

This section showed how providing context and culturally relevant ADRD information that is accessible is requested by the Black American community.

#### Theme 3: Physical Places and Existing Social Networks Are Necessary to Spread Lola Among the Community

This last theme is related to the importance of using physical community-oriented places and pre-existing social networks to spread technological tools among the Black American community. Our participants suggested community-oriented ways to advertise these health-related technologies to be adopted and used by the community. Most of the participants mentioned the importance of doing demonstrations of the Lola app in physical community places such as a town hall.

I think if they’re going to implement this process, then they should have maybe a town hall meeting to go through the process have a big screen. The people that are interested in finding out more than they’ll do it step by step. So I think that will be a demonstration we’re having at a community center or, or even in a building like this, and advertise that we have a new application now called Lola that will help.

P1

Finally, participants mentioned how, in the advertisement of the Lola app, it is important to leverage pre-existing social networks within the community, such as relying on word of mouth and showing how people within the community are using Lola.

More information to let us know, what makes Lola different from the other? That will make me want to go to Lola more. And that could cover word of mouth, that could cover the internet, having more of these to get people involved to know more, maybe part of the marketing or the draw from my community would be Lola specifically, or is more strategic as far as getting information out to Black communities.

P9

This section highlighted how physical locations where the community gathers and existing social networks are vital to promote increased use of Lola, as word-of-mouth marketing is effective when performed by trustworthy individuals.

## Discussion

### Principal Findings

Deployment of generative AI in the health care domain offers the promise of innovation and expands the potential of equitable health technologies. However, erroneous design and development of generative AI could increase bias and potential harm toward historically marginalized and underserved communities. Including underserved populations in the design and development of a technological intervention has been shown to increase the opportunity for representation and inclusivity [27-31]. Following this line of research, our study investigated the experiences of Black American caregivers using a generative AI tool to seek ADRD-related information (such as symptoms, community resources, financial aid).

This is the first study that specifically addresses the needs, demands, and challenges of this specific population from the perspective of caregivers of individuals with ADRD. An in-depth description of the 3 key design implications to create an inclusive and culturally relevant AI-supported platform for health will follow.

### Fostering Inclusivity Through Multimodality

We observed how Black American caregivers expressed the need for multimodality of interaction to equitably access what generative AI tools have to offer. Indeed, they expressed the importance of being able to “talk with the tool” and hear back (eg, read content out loud) in the fashion of a conversational voice assistant. Several participants’ insights suggested a preference toward multimedia, emphasizing the auditory and visual senses and providing modes (speaking, reading, and listening) to support these interaction styles. These implications may originate from their day-to-day time constraints, requiring short and efficient interactions.

Previous work addressed the efficiency of using multimodal systems that provide health care. Linders et al [59] created a multimodal health assistant to provide health information. Soubutts et al [60] used a multimodal device to support the needs of households for health and care purposes during COVID-19. The need for multimodal interactions for memory aid systems has been also highlighted [61].

Additionally, multimodality can guarantee a larger audience who will interact with the system [62], thereby allowing people with low vision and low hearing to access the system and seek ADRD health-related information. Within the landscape of designing for inclusive and equitable computing, our study took the approach of multimodal interaction. Our findings emphasize the need to tailor technology to accommodate different interaction styles. This can be achieved by designing multimodal technologies that can meet the needs of differently abled users.

### Answering the Call for Context and Culture-Specific ADRD Information

Our findings reflected the caregivers’ need for contextually and culturally relevant ADRD information that is specific to their communities or personalized based on their needs and preferences. They indicated their need for lifestyle tips and actionable tools for ADRD rather than only medical information in the system. They also did not want to be offered only general ADRD medical information; rather, they wanted ADRD-related information specific to their community, local resources, and geography. Locality and cultural relevance emerged as 2 of the most important factors influencing the perceived usefulness of the information sought. This is in line with previous studies [63,64] showing that providing community-centered information can increase the acceptance and adoption of generative AI tools. Compared with the use of OHC, which provides general emotional and social support, as identified by previous studies [49-51], our study reveals the need for specialized information on health care. Our participants expressed their need for instrumental and informational support specific to their social and cultural context as well as geographic location. This finding complements the benefits of OHCs to meet all the needs of caregivers including instrumental and information support.

Furthermore, our participants wanted to be sure that the information provided was accessible to people with low literacy skills within their own communities. This is in line with the collectivist nature of African American communities observed in a prior study on technology use [30], wherein, for a technological intervention to be accepted, it must be inclusive of all the members of the Black American community.

### Keeping Not Only the People But Also the Community in the Loop

Finally, our study offers a novel contribution in generative AI research investigating how generative AI tools can be advertised and spread among the Black American community. To our knowledge, this is the only study that has investigated this aspect of generative AI use and adoption. Indeed, low technology adoption is a striking problem within Black American communities, especially among people with low economic status, which prevents them from using online tools to access health information and improve their health literacy [65]. However, low adoption does not arise from the lack of digital tools but also from a skepticism toward digital tools [66]. Privacy is also a crucial concern, as Black American populations tend to be more skeptical toward the use of technology based on a fear of privacy intrusions and breaches of privacy [41,63]; thus, generative AI–based technologies for this population should foster a transparent and reliable way of handling user privacy.

Some studies [35,66-68] have investigated how faith-based organizations can be important for health dissemination and assisting Black American adults seeking health information. Our study expands on these previous studies by exploring the strategies suggested by our participants, which could be used to foster generative AI adoption and its use among their communities.

Hence, although most studies have focused on programs designed to disseminate information based upon the creation of seminars and physical events, our study provides evidence of how physical organizations (faith-based organizations, public as well as medical organizations) could be strategic actors in fostering the adoption and use of a generative AI tool for ADRD. Additionally, given that these populations have limited resources and opportunities to interact with AI systems and often have difficulties accessing any technology (eg, software, hardware, internet) due to financial and infrastructural challenges [39], incorporating these physical organizations is significant. Building trust and familiarity by involving faith-based organizations and communities in the dissemination of the tool, as well as in the tool’s use, facilitates not only dissemination but also the ability to break the barrier of skepticism toward health technology, by showing the community how it could embrace the tool.

Finally, ethical implications and concerns must be addressed in the discussion of the findings, even though ethical concerns did not explicitly emerge in this study as its focus was on usability, accessibility, cultural relevance, and adoption. However, it is crucial to discuss how topics such as consent, privacy, and bias can impact the use of generative AI for an underrepresented community. As suggested by previous studies, ethical guidelines committed to social good and human well-being should be developed and followed by AI system developers [32]. Consent and data management must be at the core of every generative AI system, allowing users to be in full control of their own data and their interaction with the tool. Furthermore, it is critical that users who interact with or provide data for AI systems fully understand what they are consenting to. This is especially important for populations with lower literacy rates, for whom the clarity and accessibility of consent forms must be carefully considered.

### Limitations

This study is subject to several limitations. First, the sample characteristics are not representative of the broader population. The age range of our participants may not adequately capture the unique challenges faced by younger caregivers who juggle multiple responsibilities alongside caregiving. Future studies should aim to recruit a more diverse sample to better understand the challenges different demographics face in accessing health information for caregiving.

Furthermore, our participants demonstrated a high level of education, with the majority (12/15, 80%) having some college education. This could have influenced our findings, potentially skewing the results toward those with higher educational backgrounds. We recommend future research include Black American individuals with lower literacy levels to better understand their specific needs and how they engage with AI-generated health technologies.

Last, the user experience testing sessions for this study were conducted outside of the participants’ daily environments, which may not accurately reflect the day-to-day challenges they might encounter when interacting with Lola. This limitation could restrict the scope of identified problems. Future studies should consider conducting tests in settings and scenarios that more closely mimic real-life conditions to gain deeper insights into the tool’s practical efficacy and user engagement.

Apart from the opportunities already identified, our study opens the research space on the intersection between generative AI and underrepresented populations living in the United States. One possibility could be investigating the effectiveness of tailoring ADRD content to the community’s values; hence, future studies could quantify the effectiveness of such interventions on user engagement as well as on ADRD-related health literacy. Second, future studies could investigate the impact of different interaction modalities on user engagement, thus understanding whether multimodality is preferred over solely having the opportunity of interacting with one modality, and explore the potential benefit of using different modalities over others in groups with various needs.

### Conclusions

This study adds to the expanding field of research focused on deploying generative AI to improve health literacy. More specifically, the emphasis of this study was on improving the ADRD-related health literacy of Black American informal caregivers, who often are solely responsible for individuals with ADRD. Our objective in testing our intervention, Lola, was to identify the specific aspects of our design that need to be tailored and modified. This approach aims to develop a tool that can genuinely assist this population, enhance their ADRD-related health literacy, and support them in their caregiving responsibilities.

Our findings showed that ADRD-specific content should be made contextually and culturally relevant to be perceived as useful by informal caregivers, thereby including information on local, geographically dependent resources. This shows how generative AI designed to improve ADRD-related health literacy should be highly personalized and dependent upon the user’s social and cultural context. Additionally, concerning the interactions, relying on multimodality can contribute to creating an accessible tool for informal caregivers, by offering them multiple and diverse interaction strategies. Once again, this finding indicates the need for a generative AI tool that can adapt to the users’ physical and cognitive needs and demands.

Finally, through active collaboration and community engagement, this study provides evidence of the importance of including the users and population of interest in the evaluation of a design as a way to guarantee that generative AI technologies will benefit all individuals within society, even underrepresented communities such as Black American populations.

## Abbreviations

ADRD: Alzheimer disease and related dementias
AI: artificial intelligence
GPT: generative pretrained transformer
LLM: large language model
OHC: online health community
